# BBB-penetrating codelivery liposomes treat brain metastasis of non-small cell lung cancer with EGFR^T790M^ mutation

**DOI:** 10.7150/thno.42234

**Published:** 2020-05-15

**Authors:** Weimin Yin, Yuge Zhao, Xuejia Kang, Pengfei Zhao, Xuhong Fu, Xiaopeng Mo, Yakun Wan, Yongzhuo Huang

**Affiliations:** 1 State Key Laboratory of Drug Research, Shanghai Institute of Materia Medica, Chinese Academy of Science, Shanghai 201203, China; 2 Institute of Pediatrics of Children's Hospital and Biomedical Science, Fudan University, Shanghai 200032, China; 3 Nanchang University College of Pharmacy, 461 Bayi Rd, Nanchang 330006, China; 4 NMPA Key Laboratory for Quality Research and Evaluation of Pharmaceutical Excipients

**Keywords:** brain targeting delivery, tumor-associated macrophage, non-small cell lung cancer (NSCLC), drug resistance, tyrosine kinase inhibitors (TKI), EGFR T790M mutation

## Abstract

EGFR TKI therapy has become a first-line regimen for non-small cell lung cancer (NSCLC) patients with EGRF mutations. However, there are two big challenges against effective therapy--the secondary EGFR mutation-associated TKI resistance and brain metastasis (BMs) of lung cancer. The BMs is a major cause of death for advanced NSCLC patients, and the treatment of BMs with TKI resistance remains difficult.

**Methods:** Tumor-associated macrophages (TAM) is a promising drug target for inhibiting tumor growth, overcoming drug resistance, and anti-metastasis. TAM also plays an essential role in regulating tumor microenvironment. We developed a dual-targeting liposomal system with modification of anti-PD-L1 nanobody and transferrin receptor (TfR)-binding peptide T12 for codelivery of simvastatin/gefitinib to treat BMs of NSCLC.

**Results:** The dual-targeting liposomes could efficiently penetrate the blood-brain barrier (BBB) and enter the BMs, acting on TAM repolarization and reversal of EGFR^T790M^-associated drug resistance. The treatment mechanisms were related to the elevating ROS and the suppression of the EGFR/Akt/Erk signaling pathway.

**Conclusion:** The dual-targeting liposomal codelivery system offers a promising strategy for treating the advanced EGFR^T790M^ NSCLC patients with BMs.

## Introduction

An era of chemo-free is approaching in non-small cell lung cancer (NSCLC) therapy. As a case point, molecular targeting therapy using epidermal growth factor receptor (EGFR) tyrosine kinase inhibitors (TKI) has changed the landscape of NSCLC clinical practice and substituted chemotherapy as a first-line treatment for patients with EGFR mutations 1. EGFR mutation is the most well-established driver mutation and the most important drug target in NSCLC that comprises approximately 85% of all lung cancer, and it was found a high EGFR mutation frequency (51.4% overall) in the tumors from Asian NSCLC patients 2. Although there has been a breakthrough in TKI therapy, a formidable challenge is drug resistance that typically develops after a median of 9-14 months of TKI treatment 3. For example, gefitinib (Gef) is a standard treatment for the NSCLC patients harboring the EGFR L858R mutation, but over 60% of the patients receiving Gef therapy would develop secondary mutation T790M (threonine substituted by methionine at amino acid position 790) and cause drug resistance 4.

Moreover, metastases account for >70% of deaths of the patients with advanced-stage NSCLC 5, and brain metastasis (BMs) occurs in approximately 33% of the patients with EGFR mutation 6. Much worse, the incidence of BMs has been increasing in the recent decade and is a major cause of death for NSCLC patients 7. The BMs pharmacotherapy remains difficult because of a delivery problem caused by the blood-brain barrier (BBB). Osimertinib (Osi), a third-generation EGFR TKI and now a first-line drug for NSCLC, is effective to the brain metastatic EGFR^T790M^-positive NSCLC patients. Yet, it can also acquire drug resistance (C57S mutation) rapidly 8. Therefore, it is highly desired to develop an effective strategy to overcome drug-resistant BMs.

Tumor microenvironment is associated with cancer progression, metastasis drug-resistance, and immune evasion 9. Our previous works demonstrated a therapeutic strategy by remodeling tumor microenvironment was effective to reverse EGFR^T790M^-associated drug resistance in a subcutaneous lung tumor model 10, 11. However, the feasibility of treating EGFR^T790M^-mutated BMs is unknown. The BBB is the first barrier that rejects most drugs to enter the brain 12. Several strategies of overcoming the BBB have been identified, including cell-penetrating peptide-mediated BBB penetration, opening BBB, nose-to-brain delivery, and dual-targeting delivery 13-16.

To address both the delivery and therapeutic challenges of TKI-resistant BMs, we developed the dual-targeting liposomes modified with anti-PD-L1 nanobody (Nb) and a transferrin receptor (TfR)-binding T12 peptide for mediating BMs-targeting drug delivery. Programmed cell death ligand 1 (PD-L1) is an immune checkpoint protein that overexpresses not only on the cancer cells but also tumor-associated immune cells; for example, tumor-associated macrophages (TAM) highly express PD-L1 and lead to immune-suppression 17. Moreover, high expression of PD-L1 was also found in tumor vessel epithelial cells 11. Nbs are a class of single-domain antibody fragments with a benefit of small size, and anti-PD-L1 Nbs could be advantageously utilized as a targeting ligand for nanocarriers. T12 peptide can mediate the BBB penetration and brain tumor delivery 18, with a binding site on TfR different from that of transferrin, thereby avoiding the binding competition with endogenous transferrin 19. Therefore, it was expected that the dual-targeting strategy could be able to overcome BBB and direct to BMs and the tumor microenvironment to improve the treatment.

TAM is an essential component in the tumor microenvironment, representing up to 50% of the tumor mass, and is closely associated with tumor growth, angiogenesis, metastasis, and drug resistance 20. TAM is characterized by two subtypes—the anti-tumor M1 and pro-tumor M2 21. Therefore, TAM repolarization from M2 to M1 is a promising therapeutic strategy and a useful method for remodeling tumor microenvironments 22, 23.

Simvastatin (SV) is a commonly used cholesterol-lowering drug. Epidemiologic evidence also demonstrates the antitumor effect of SV 24. We previously revealed that the SV-based treatment could repolarize the TAM from M2 to M1 phenotype 11, 25, and re-sensitize the T790M mutated cells to gefitinib therapy 14. Hence, on the basis, we developed a T12/PD-L1 Nb-dual modified liposomal system for SV/Gef brain codelivery to overcome the two formidable barriers in NSCLC treatment--the BMs and drug resistance. Yet, there is no EGFR^T790M^-mutated H1975 brain metastasis model currently available. In this study, we used an H1975 intracranial xenograft model for a proof-of-concept study.

## Results

### Characterization of the T12 peptide and anti-PD-L1-modified liposomes (T12/P-Lipo)

T12 peptide (sequence: CGGGTHRPPMWSPVWP) was conjugated to DSPE-PEG_2000_-Mal via the sulfhydryl-maleimide reaction. The ^1^NMR and the MALDI-TOF MS confirmed the product of DSPE-PEG-T12 (Figure S1A-B). The anti-PD-L1 Nbs were prepared according to the procedures in our previous study 11.

**Figure 1:**
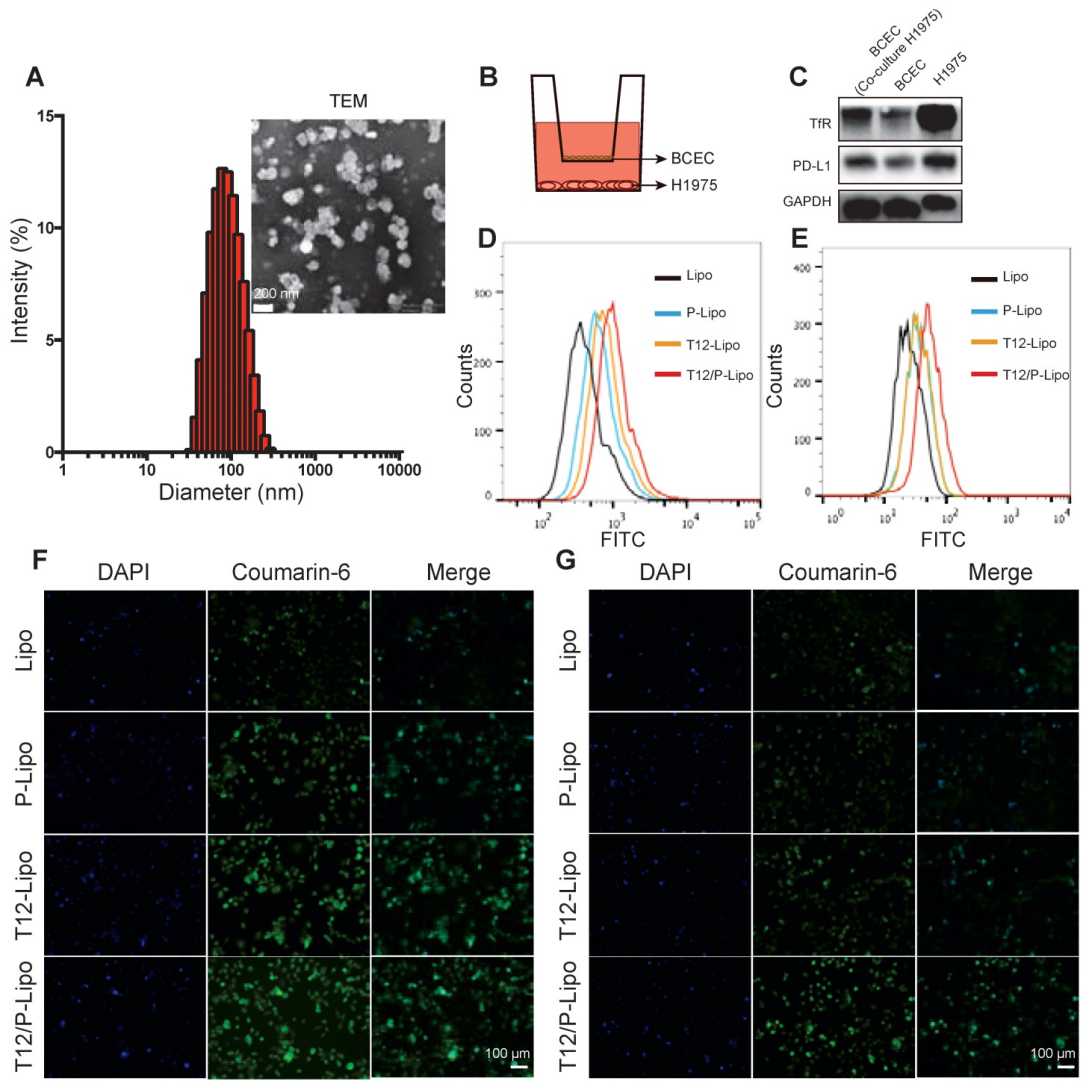
The characterizations of the T12/P-Lipo. (A) The particle size and TEM of the T12/P-Lipo. (B) Transwell co-culture model. (C) The targeting receptors (TfR and PD-L1) in the BCEC w/ or w/o co-culture of H1975 cells, as well as in the H1975 cells alone. (D) Uptake efficiency of Lipo, P-Lipo, T12-Lipo, and T12/P-Lipo in the H1975 cells. (E) Uptake of Lipo, P-Lipo T12-Lipo and T12/P-Lipo in the H1975 cells after penetration through the BCEC monolayer at the Transwell. (F) Fluorescence images of the intracellular Lipo, P-Lipo T12-Lipo, and T12/P-Lipo in the tumor cells. (G) Fluorescence images of the intracellular Lipo, P-Lipo, T12-Lipo, and T12/P-Lipo in the H1975 cells after penetration through the BCEC monolayer at the Transwell.

The particle size of the T12/P-Lipo was around 153 nm and the zeta potential about -27 mV (Figure 1A, S2C, Table S1). The drug-encapsulation efficiency and drug-loading capacity of the liposomes were shown in Table S1, and the dose ratio of Gef and SV in the liposomes was 1:1 (w/w). The liposomes exhibited good stability in PBS containing 10% FBS (Figure S2A), and Gef and SV were released in an identical pattern (Figure S2B).

The trans-epithelial penetration study was used to evaluate the BBB-permeability of the T12/P-Lipo using a Transwell culture model, in which the brain capillary endothelial cells (BCECs) were in the upper chamber and the H1975 NSCLC cells in the lower chamber (Figure 1B). The expressions of PD-L1 and TfR in the BCECs were upregulated when co-cultured with the H1975 cells (Figure 1C). It suggested that both PD-L1 and TfR were the potential delivery targets in the brain tumor vessel endothelial cells. Furthermore, the H1975 cells also highly expressed the TfR and PD-L1 protein (Figure 1C). The uptake of T12/P-Lipo by the H1975 cells was 1.6-fold greater than the PD-L1 Nbs-modified liposomes (P-Lipo), 1.3-fold than T12-Lipo, and 2.5-fold than non-modified liposomes (Lipo) (Figure 1D). The fluorescence imaging results further confirmed the high uptake efficiency of T12/P-Lipo in H1975 tumor cells (Figure 1F). In the Transwell study, after penetration through the BCEC monolayer, the uptake efficiency of T12/P-Lipo in the cancer cells was 1.52-fold greater than P-Lipo, 1.5-fold than T12-Lipo, and 2.13-fold than Lipo (Figure 1E). Furthermore, the fluorescence images also confirmed the enhanced BBB-penetrating ability of the T12/P-Lipo and the subsequent uptake by the H1975 cells (Figure 1G). Therefore, this dual-modified liposomal system exhibited a potential for brain drug delivery.

### M2→M1 repolarization and anti-angiogenesis

**Figure 2:**
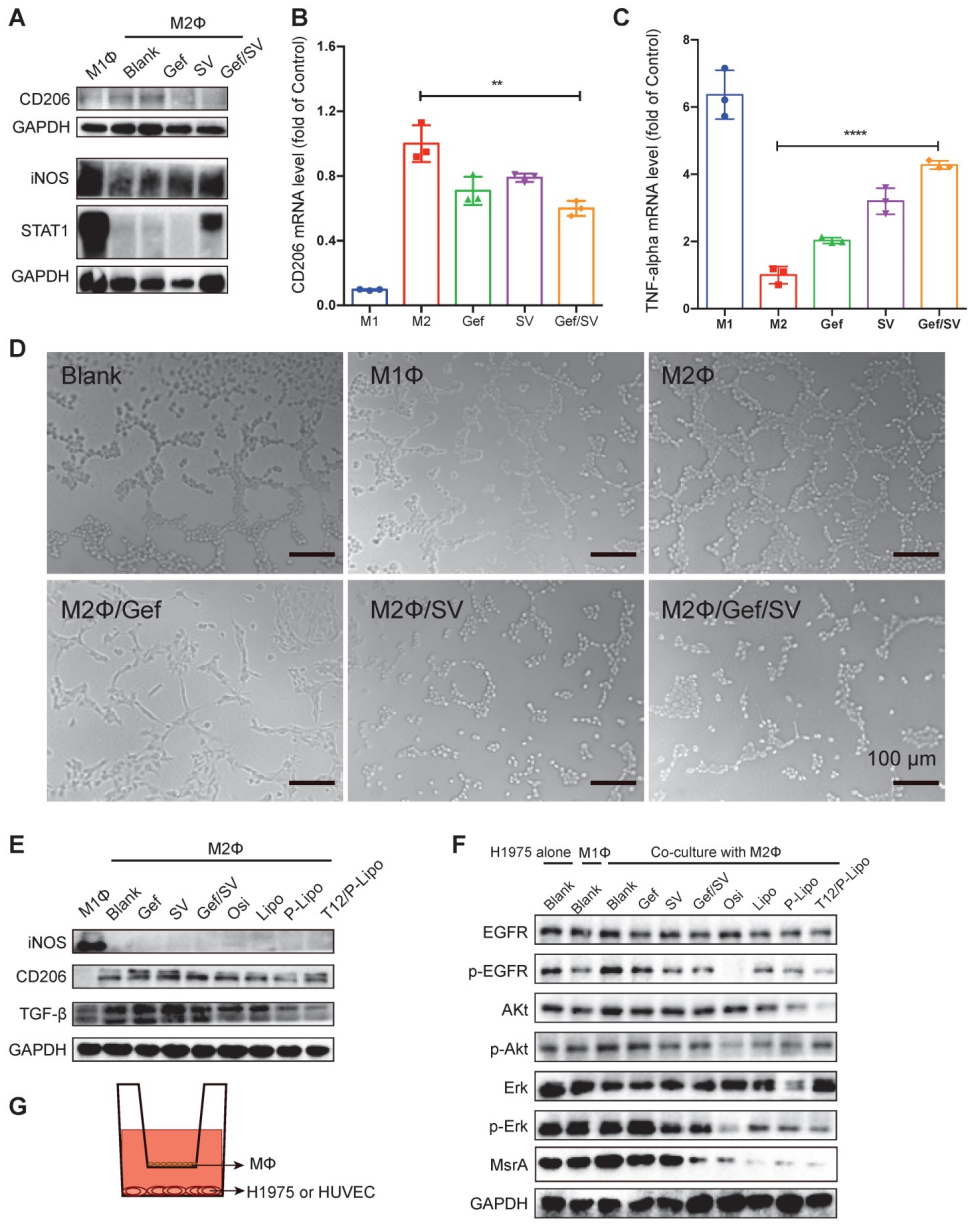
The antitumor mechanism study. (A) CD206 and STAT1 or iNOS expression in M1 and M2Φ. (B) CD206 and (C) TNF-α mRNA level in M2Φ after drug treatment. M1Φ and M2Φ without treatment were used as controls. (D) Anti-angiogenic ability indicated by the HUVEC tube formation. (E) The levels of iNOS, CD206, and TGF-β in M2Φ post-treatment. (F) The levels of phosphorylated EGFR/Akt/Erk and MsrA in H1975 tumor cells with co-culture with M2Φ. (G) The Transwell co-culture system of macrophages and HUVEC (or H1975 tumor cells).

After treatment, the results showed the downregulation of CD206 (an M2-associated marker) and upregulation of STAT1 or iNOS (an M1-associated marker) (Figure 2A). The qPCR results also revealed that the mRNA level of CD206 was decreased while TNF-α (an M1-associated cytokine) was increased (Figure 2B-2C). These data demonstrated that combinatory SV/Gef repolarized M2 to M1 subtype.

TAM plays an essential role in promoting tumorigenesis through angiogenesis and metastasis 17. Our results revealed that M1Φ inhibited the tube formation of the human umbilical vein endothelial cells (HUVEC). By contrast, M2Φ accelerated the HUVEC tube formation, which, however, was remarkably inhibited if treating M2Φ with Gef/SV (Figure 2D). It could account for the reversal of M2Φ polarization by the Gef/SV-based treatment.

Transforming growth factor-β (TGF-β) is a major protumor cytokine secreted by TAM and the primary driven factor to induce epithelial-mesenchymal transition (EMT) and EMT-associated metastasis and drug resistance 23, 26. Our results showed that the T12/P-Lipo treatment can repolarize M2, evidenced by the downregulation of CD206 and TGF-β (Figure 2E).

The MTT assay revealed that the drugs at the tested concentrations were safe to macrophages (Figure S3A) and the Q-PCR assay also suggested that the increasing drug concentration facilitated TAM reversal (M2→M1) (Figure S3B), instead of inducing TAM depletion.

Furthermore, to mimic the tumor microenvironment, the Transwell co-culture model was used to evaluate the MΦ repolarization effect, with the upper chamber of TAM (e.g., M1Φ and M2Φ) and the lower chamber of H1975 cells. The results showed that the M1Φ co-culture induced the inhibition of EGFR signaling in the H1975 cells whereas the M2Φ co-culture upregulated EGFR expression (Figure 2F). However, the combined drugs and liposome treatment significantly reversed the M2Φ effect and suppressed EGFR/Akt/Erk phosphorylation, and the T12/P-Lipo showed the highest efficiency (Figure 2F). Methionine residues in the intracellular proteins are very susceptible to reactive oxygen species (ROS), resulting in oxidation and formation of methionine sulfoxide residues, but this modification can be reversed by methionine sulfoxide reductase (MsrA) 27. We previously demonstrated that the downregulation of MsrA contributed to the decrease in T790M 10. Our results showed that MsrA was downregulated by the T12/P-Lipo treatment (Figure 2F), which could facilitate the reversal of T790M-associated resistance.

### In vitro antitumor study

**Figure 3:**
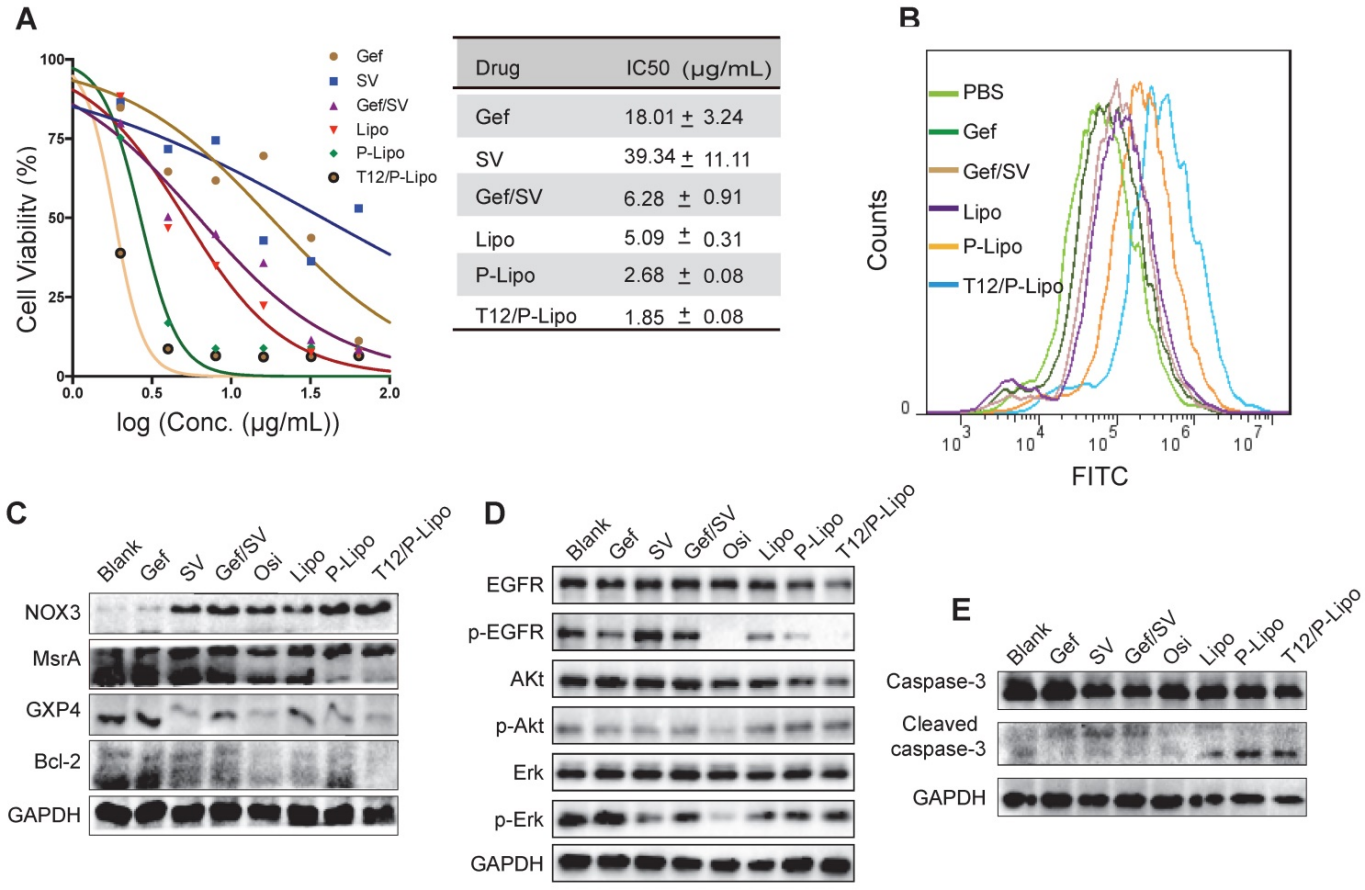
The in vitro antitumor study. (A) Antiproliferation ability in H1975 cells. (B) ROS levels in H1975 cells. (C) The NOX3/MsrA/GPX4/Bcl-2 expression in H1975 cells. (D) Downregulation of the phosphorylated EGFR/p-Akt/p-Erk. (E) Upregulation of the cleaved caspase 3.

The EGFR^T790M^ H1975 NSCLC cells were highly resistant to Gef (Figure 3A) and SV showed minor cytotoxicity to the cancer cells. By contrast, the combined Gef/SV yielded a synergistic antitumor effect (CI = 0.515) and overcame drug resistance in the H1975 cells; the activity was further enhanced by using the liposomes, showing the IC_50_ of 1.85 μg/mL for the T12/P-Lipo (Figure 3A). The results revealed the ability of the T12/P-Lipo to reverse the T790M mutation-associated drug resistance.

ROS is a critical regulator of protein kinase 28. Methionine is an oxidation-sensitive amino acid, which is commonly involved in the post-translation modification of proteins in the cells. The methionine in the mutation site of EGFR^T790M^ was sensitive to oxidation, and the enhanced ROS can promote EGFRT^790M^ degradation 29. Our results revealed that the T12/P-Lipo significantly increased the ROS level in the cancer cells (Figure 3B). The nicotinamide adenine dinucleotide phosphate (NADPH) oxidases (NOX) were responsible for ROS generation 30. The EGFR^T790M^ level can be regulated by redox balance between NOX3 and MsrA, and the upregulated NOX3 and downregulated MsrA facilitated EGFR^T790M^ degradation 31. In addition, the antioxidant enzyme glutathione peroxidase 4 (GPX4) plays an important role in regulating cellular redox status too 32. The suppression of GPX4 can cause an increased ROS level 33. The Western blotting results showed that the T12/P-Lipo increased the NOX3 level but downregulated MsrA and GPX4 (Figure 3C). It indicated that the combination therapy regulated the NOX3/MsrA/GPX4/Bcl-2 axis and cellular redox to reverse the EGFR^T790M^-associated drug resistance.

EGFR/Erk/Akt is an important growth signaling axis of the cancer cells. The combinatory Gef/SV inhibited the p-EGFR/p-ErK/p-Akt pathway (Figure 3D). Furthermore, the cleaved caspase-3, an apoptotic protein, was increased in the combination therapy groups such as T12/P-Lipo (Figure 3E), indicating the efficient induction of apoptosis of the H1975 cells, as potent as the positive control osimertinib (Osi). Accordingly, Bcl-2, an anti-apoptotic protein that is negatively regulated by the elevating ROS 34, was decreased after treatment with T12/P-Lipo and other combination groups (Figure 3C).

These results revealed that the mechanisms of Gef/SV combination were associated with the increased ROS level in the H1975 cells, thereby regulating the ROS/NOX3/MsrA/GPX4/Bcl-2 axis, suppressing EGFR/Erk/Akt phosphorylation and inducing cell apoptosis, and eventually reversing drug resistance.

### The liposomes biodistribution study

**Figure 4:**
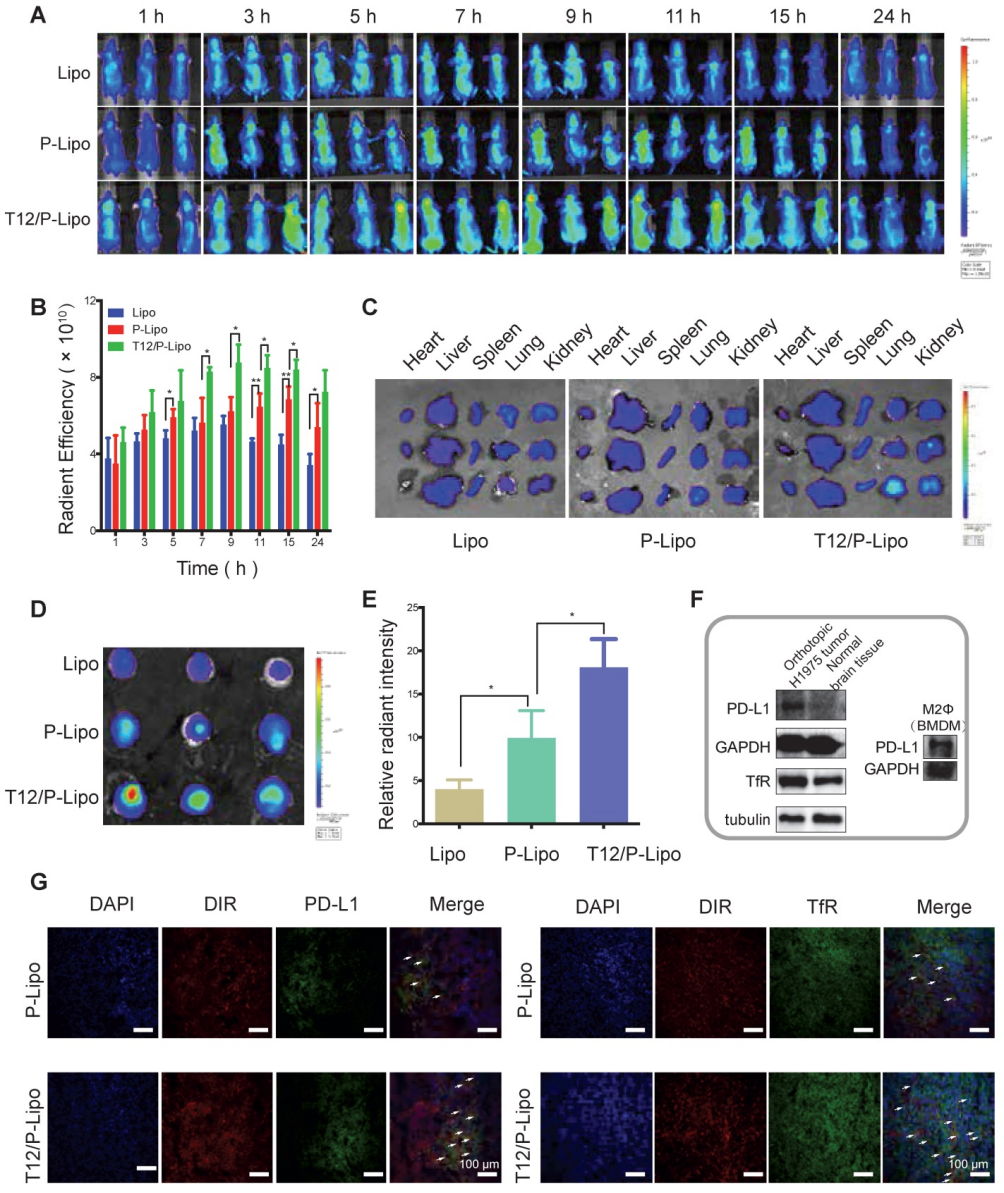
Biodistribution study of T12/P-Lipo. (A) The intratumor accumulation of T12/P-Lipo. (B) In vivo radiant efficiency of ROIs at tumor sites. (C) In vitro radiant efficiency of the organs. (D) Radiant efficiency of the dissected brain tissues. (E) Ex vivo relative radiant intensity in the brains. (F) The expression of PD-L1 and TfR in the BMs and PD-L1 in M2Φ. (G) Immunofluorescent co-localization (the arrows) of the DIR-containing liposomes and PD-L1 or TfR.

BMs-targeting delivery efficiency was investigated. The fluorescence at the brain tumor site was observed within 1 h and was gradually increased. The animals receiving the T12/P-Lipo showed the highest tumor accumulation that reached the maximum around 9 h (Figure 4A-4B). The fluorescent signal in the dissected tumors was found highest in the T12/P-Lipo group too, but there was no significant difference among the normal organs observed by ex vivo imaging (Figure 4C-E). It demonstrated that PD-L1 Nbs and T12 peptide enhanced BMs-targeting delivery efficiency.

Moreover, the intratumoral distribution of T12/P-Lipo was investigated by immunofluorescence staining. The T12/P-Lipo distribution was largely overlapped with the PD-L1 or TfR staining, supporting the delivery mechanism of PD-L1 Nbs and transferrin-facilitated dual-targeting delivery (Figure 4G). Furthermore, the Western blotting results showed that the glioma tissues highly expressed PD-L1 and TfR compared to the normal brain tissues, and the M2Φ also overexpressed PD-L1 (Figure 4F). Therefore, it indicated the multiple targeting function of T12/PD-L1 for mediating brain tumor delivery and M2Φ delivery.

The Lipo and T12/P-Lipo showed similar pharmacokinetic profiles in the female Balb/c, with the blood circulation half-life (t_1/2_) of 4.97 and 5.9 h, respectively (Figure S2D).

### In vivo treatment study

**Figure 5:**
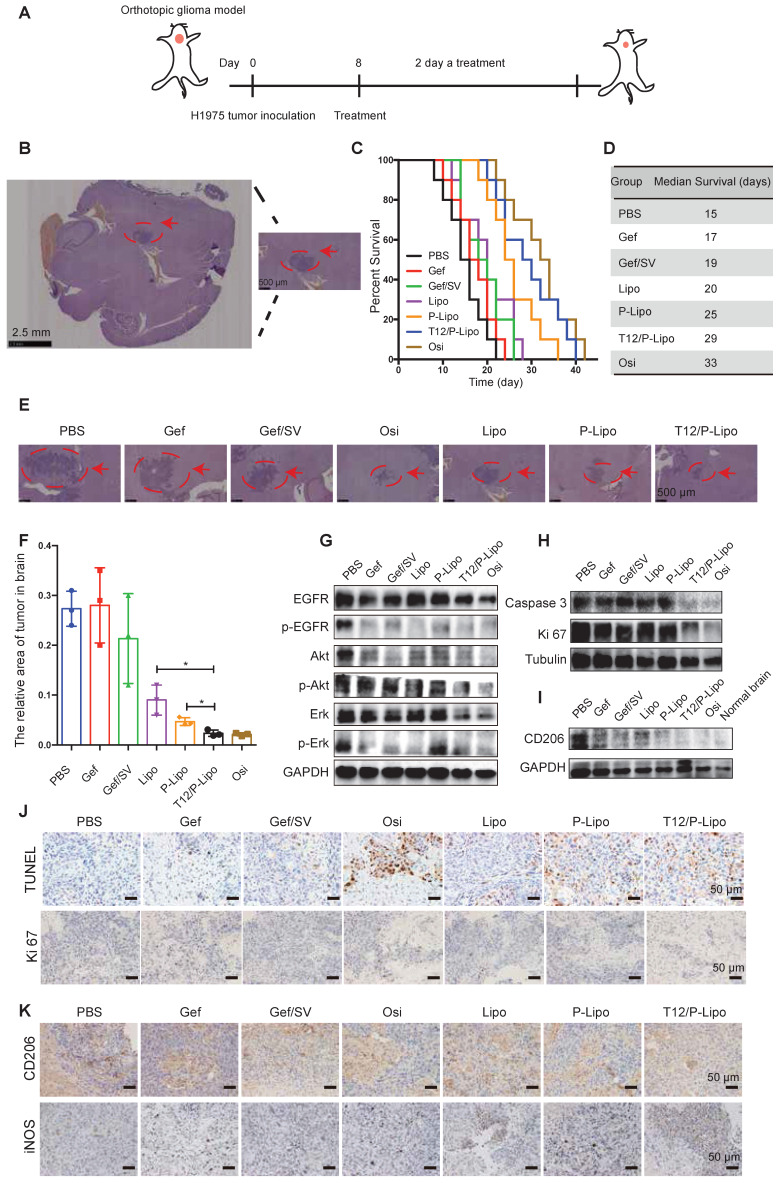
The treatment efficacy in the BMs model of H1975 NSCLC. (A) The treatment regimen. (B) The tumor location in brain tissue (H&E result). (C) The survival curves. (D) The median survival. (E) The histological examination of the brain tissues with tumor xenografts after treatment. (F) Statistical analysis of tumor regression after treatment. (G) Downregulation of p-EGFR/p-Akt/p-Erk. (H) Downregulation of caspase 3 and Ki 67. (I) The M2-associated CD206 expression. (J) The enhanced apoptosis (TUNEL staining) and reduced proliferation (Ki 67 staining) (brown color) in tumors. (K) M2-associated marker CD206 and M1-associated marker iNOS staining after treatment.

The in vivo anti-tumor efficacy was investigated using the BM mouse model developed by intracranial transplant of the H1975 NSCLC cells (Figure 5A). The immunohistochemistry (IHC) staining indicated the brain tumor area (Figure 5B). The median survival time was 29, 25, and 20 days for the T12/P-Lipo, P-Lipo, and Lipo groups, respectively; T12/P-Lipo showed the enhanced treatment efficacy than other liposomal groups (Figure 5C-D). The T12/P-Lipo group was comparable to the positive group of Osi in terms of overall survival (P = 0.396) (Table S8). The tumor regression after treatment was observed by histological examination (Figure 5E-F, Table S8), which further demonstrated the better efficacy of T12/P-Lipo over other liposomal groups. The results indicated the promise of the dual-targeting delivery for improving brain tumor therapy and overcoming drug resistance in the BMs of lung cancer. The findings provided an alternative via a mechanism of tumor environment modulation, other than the current osimertinib-based molecular targeted therapy.

The Western blotting results showed the strong inhibition of the tumor growth signaling pathway of EGFR/Erk/Akt by the T12/P-Lipo (Figure 5G). It should be mentioned that the tumor tissues contain not only tumor cells but also various stromal cells and immune cells. Therefore, it could cause a difference in EGFR/Erk/Akt levels between the cellular and animal results. However, the primary trend was similar. An extensive degree of apoptosis was elicited in the BMs of the T12/P-Lipo group (Figure 5H, 5J). Ki-67 is a cellular proliferation biomarker, and the Ki67 staining result further confirmed the improved treatment efficacy in the T12/P-Lipo group (Figure 5H, 5J).

The TAM repolarization (M2→M1) was examined by immunohistochemistry and Western blotting assay; the T12/P-Lipo treatment decreased the level of M2-associated CD206 but upregulated the M1-related iNOS (Figure 5I, 5K).

**Figure 6:**
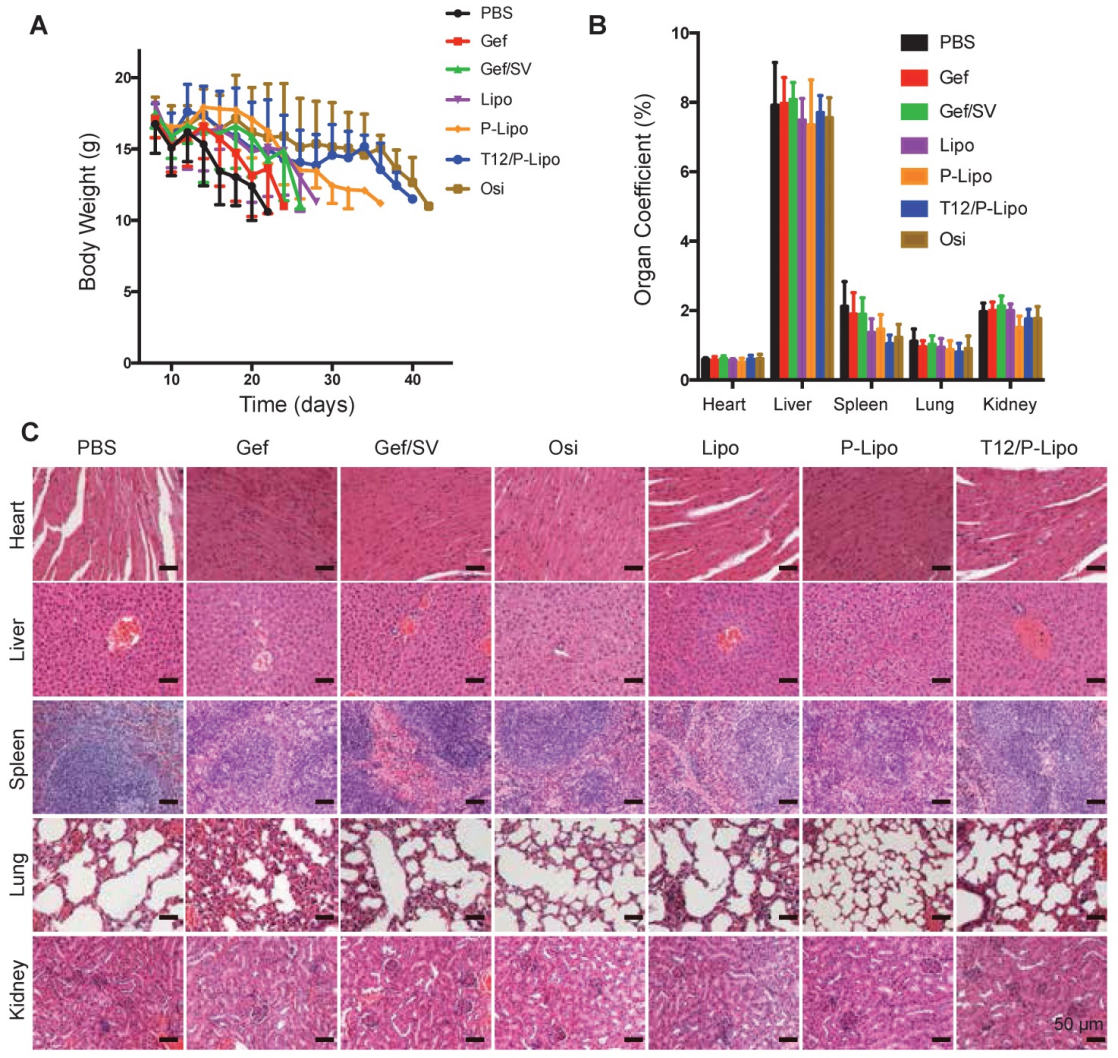
Preliminary safety examination. (A) Bodyweight changes. (B) Organs coefficient. (C) Histological examination of major organs.

Owing to the high malignancy and aggressive progression of the intracranial metastasis of NSCLC H1975 and the consequent poor appetite, all the animals lost weight at the late experimental stage; yet, the T12/P-Lipo group exhibited the improved condition among the tested groups (Figure 6A). Compared to the saline control, all the treatment groups showed no significant difference in the organ coefficients (Figure 6B). Importantly, no pathological changes in the major organs were seen in the histological examination (Figure 6C), indicating the biosafety of this nanomedicine.

## Discussion

EGFR^T790^ secondary mutation is a major cause for failure of Gef-based molecular targeting therapy. Although Osi has been approved to treat the patients with EGFR L858R^+^/T790M^+^ mutation, the new drug-resistant C797S isoform mutation rapidly occurs and results in treatment failure 35. It thus implies the challenge of overcoming the mutated EGFR-associated drug resistance through the conventional “key (TKI) and lock (EGFR)” pattern, because there is a hard race that it takes 1-2 decades to develop a new TKI drug but the mutation-associated resistance occurs merely within 1-2 years with TKI treatment.

Tumor microenvironment and TAM have been emerged as a promising target for overcoming drug resistance and metastasis. Our previous works demonstrated that EGFR^T790M^ level can be regulated by tumor oxidative microenvironments by repolarization of TAM 10. The molecular mechanism was related to the oxidative degradation of methionine 31. We also found that the tumor microenvironment remodeling (e.g., anti-angiogenesis and TAM repolarization) also contributed to the reversal of EGFR^T790M^-associated drug resistance by using a SV/Gef codelivery liposome 11. To apply the treatment strategy via tumor microenvironment modulation, we developed a BBB-penetrating delivery strategy in this present work by using a new design of dual-targeting liposome modified with T12 peptide and anti-PD-L1 nanobody.

Apart from drug resistance, BMs is a leading cause of death for advanced NSCLC patients 36. Therefore, the mutation-associated drug-resistant BMs imposes the double challenges for TKI treatment. Our dual targeting design can serve as a “two-birds-one-stone” strategy by simultaneously acting on both the BMs and TAM. The T12/P-Lipo could efficiently target the BMs and TAM via the TfR and PD-L1 receptor. The SV-based combination therapy can “re-educate” TAM from M2 to M1 phenotype. The repolarization of TAM led to the suppression of EGFR/Akt/Erk signaling that controlled the cancer cell proliferation. Besides, the activation of M1 macrophage-mediated innate immunity played a role in arresting the NSCLC tumor growth, as we previously reported 11. Yet, the detailed mechanisms of the TAM-targeting therapy for overcoming mutation-associated drug-resistant BMs should be further investigated.

Last but not least, nanomedicine-based combination therapy provides a useful tool for drug repurposing--an old drug for a new disease indication. Due to the benefit of rapid clinical impact at a lower cost than de novo drug development, drug repurposing has become an R&D information resource and served as a next-generation drug candidate library.^37^ However, there is a gap between the in vitro test and in vivo study for combination therapy, because the combo free drugs are often not consistent with their in vivo fate 38. It thus imposes a barrier against the efficient screening for drug repurposing. In this work, we reported the application of SV for treating the drug-resistant BMs of lung cancer by using a dual-targeting liposomal codelivery system, which was able to reverse the mutation-associated drug resistance and enhanced treatment efficacy on the mice with BMs. SV has been rarely reported in the animal studies of brain tumors, presumably due to not reaching an effective concentration by a conventional dosage form. The codelivery technique provides a facile method to explore SV-repurposing combination therapy.

## Conclusion

The application of nanomedicine for treating the EGFR^T790M^-associated resistant BMs of lung cancer was not investigated yet. To address two formidable challenges (i.e., brain delivery and drug resistance) against effective therapy, we developed a T12/anti-PD-L1 Nb modified, dual targeting liposomal codelivery system for SV/Gef combination therapy. The T12/P-Lipo could efficiently penetrate the BBB and dual-target the BMs and TAM. The repolarization of TAM reversed the EGFR^T790M^-associated resistance, and the survival time of the experimental animals was significantly extended. It provides a novel and promising strategy for treating the advanced NSCLC with the drug-resistant BMs.

## Materials and Methods

### Materials

Gefitinib, simvastatin, and fluorescent dyes were obtained from Melone Pharmaceutical (Dalian, China). Osimertinib was from Selleck Chemicals (Texas, USA), and murine cytokines were from Peprotech (New Jersey, USA), and LPS from Sigma-Aldrich (St. Louis, USA). Soybean phosphatidylcholine (SPC), cholesterol, and DSPE-PEG_2000_, DSPE-PEG-NHS, and DSPE-PEG-Mal were obtained from Advanced Vehicle Technology (Shanghai, China). The derivative T12 peptide (sequence: CGGGTHRPPMWSPVWP) was synthesized by Bankpeptide Biological Technology (Hefei, China). The primary antibodies of EGFR, phospho-EGFR (Tyr1068), Erk1/2, phosphor-Erk1/2 (Thr202/Tyr204), Akt, phosphor-Akt (Ser473), VEGFR2, VEGF, caspase3, cleaved-caspase3, LC3, TGF-β, GAPDH, and galectin-3 were purchased from Cell Signal Technology (Boston, USA). The primary antibody β-Actin was obtained from Sigma-Aldrich (St. Louis, USA). The primary antibodies of CD206 and TNF-α were from Abcam (Cambridge, UK). Anti-HIF-1α was obtained from Novus (Shanghai, China) and anti-IFN-γ was from Absin (Shanghai, China). The horseradish peroxidase (HRP)-conjugated goat anti-rabbit/mouse lgG secondary antibody was obtained from Beyotime (Shanghai, China). All other reagents (analytical grade) were provided by Sinopharm Chemical Reagent Co. Ltd. (Shanghai, China).

### Cell lines

The NSCLC H1975 cells, BCEC, and HUVEC were obtained from Shanghai Cell Bank, Chinese Academy of Sciences (CAS). The H1975 cells were cultured in RPMI-1640 medium, and the HUVEC and BCEC cells in DMEM medium, both with 10% FBS and antibiotics (100 μg mL^-1^ of streptomycin and 100 U mL^-1^ of penicillin).

### Animals

Female Balb/c nude mice (5-6 weeks) were purchased from Shanghai Laboratory Animal Center, CAS. The animal protocols were approved by the Institute Animal Care and Use Committee of Shanghai Institute of Materia Medica, CAS.

### Synthesis and characterization of the T12 peptide modified DSPE-PEG_2000_

The T12 peptide (10 mg/mL in H_2_O) with a terminal cysteine was added dropwise to the DSPE-PEG_2000_-Mal solution (10 mg/mL in methyl alcohol) at a molar ratio of 3:1 and reacted under magnetic stirring for 15 h in 4°C. The DSPE-PEG_2000_-T12 conjugate was purified using a dialysis tube (MWCO 2000) and then subjected to freeze-drying. The product was characterized by 1H-NMR and MALDI-TOF mass.

### The preparation of liposomes

A standard thin film-hydration method was used. The lipid mixture (SPC, cholesterol, DSPE-PEG_2000_-NHS, and DSPE-PEG_2000_-T12 at a ratio of 30: 1.5: 1:1.5, w/w) and the drugs (Gef and SV, 1:1, w/w) were dissolved in chloroform. The optimized ratio of 1:1 (Gef/SV) was revealed in our previous work 11. The organic solvents were removed using a rotary evaporator under a vacuum condition. The thus-formed film was hydrated by using a glucose solution (5%). The suspension was subjected to water-bath ultrasound and then extruded through a 200-nm polycarbonate membrane using an extruder (Avanti Polar Lipids, Alabaster, USA). The free drugs were removed using a Sephadex G-50 column. The purified liposomes were further incubated with anti-PD-L1 Nbs at a ratio of 0.282:1 (w/w) of Nbs/DSPE-PEG_2000_-NHS for 24 h at 4°C, thus obtaining the T12/PD-L1 Nbs modified liposomes (T12/P-Lipo). The PEGylated liposomes without modification of T12 and anti-PD-L1 (termed Lipo) and the PD-L1 Nbs-modified liposomes without DSPE-PEG_2000_-T12 (termed P-Lipo) and T12-modified liposomes (T12-Lipo) were used as the controls. The theoretic ratio of dual ligands (T12/anti-PD-L1) in the liposomes was 1:1, which was based on empirical evidence in our experience on dual-targeting design and was also a common ratio in dual-targeting delivery studies 39.

### Characterizations of the Liposomes

The particle size and PDI were determined using a Zeta Size Nanoparticle Analyzer (Malvern, UK). The liposomal morphology was observed by transmission electronic microscopy (TEM). The concentration of the drugs was determined using high-performance liquid chromatography (HPLC, 1260 Infinity, Agilent technologies, USA) equipped a C18 column (250 x 4.6 mm, 5 μm, Agilent). For Gef determination, the HPLC mobile phase was water/methanol (45:55) with 0.1% trifluoroacetic acid at a detection wavelength of 330 nm, and for SV, the mobile phase was 0.1 M sodium dihydrogen phosphate buffer (pH 6.5)/acetonitrile (35:65) with 0.1% trifluoroacetic acid at a wavelength of 233 nm. The flow rate was 1 ml/min.

The encapsulation efficiency (EE%) and drug-loading capacity (DL%) were calculated as follows.

EE (%) = W_DE_ / W_T_ × 100

DL (%) = W_DE_ / w_L_ × 100

where W_DE_ was the amount of encapsulated drug in the liposomes, W_T_ was the amount of the added drug, and W_L_ was the total weight of the liposomes.

### Characterization of the liposomes

The stability of the liposomes was evaluated by dispersing the samples in PBS (pH 7.4) containing 10% FBS and the liposome size was monitored at different time points.

The in vitro drug release was assessed using a standard dialysis method (MWCO 10-14 kDa) in the PBS containing 0.5% v/v Tween 80 under a sink condition at 37°C with gentle shaking. The drug concentration was analyzed by HPLC.

### Cellular uptake assay

The H1975 cells were seeded in the 12-well plates at a density of 1 × 10^5^ cells per well and cultured for 24 h. The cells were incubated with the coumarin-6-labeled liposomes for 1.5 h, and afterward, thoroughly washed with PBS and collected for flow cytometric assay (FACS Calibur, Becton Dickinson, USA).

### Trans-epithelial transport in a monolayer transwell culture

The intact monolayer of brain capillary endothelial cells (BCECs) was used as an in-vitro culture model for preliminary evaluation of BBB penetration. In brief, the BCEC monolayer cell culture was in the upper chamber of a Transwell device (0.4-μm pore size, Corning, USA), representing an in-vitro BBB model, and the H1975 cells were cultured in the lower chamber. The coumarin-6-labeled liposomes were added to the upper chamber and incubated for 2.5 h. The H1975 NSCLC cells at the lower chamber were collected for flow cytometric assay. In addition, the cells were fixed with 4% paraformaldehyde for 20 min and stained with DAPI for fluorescent imaging (fluorescence microscope, CARL ZEISS, Germany).

### The polarization of M1Φ and M2Φ

The bone marrow-derived macrophages (BMDM) were obtained from BALB/C mice, and the M1 or M2 polarization was induced according to a protocol previously reported 11**.**

### Quantitative real-time polymerase chain reaction (qRT-PCR)

Total RNA was isolated from the macrophages with the Trizol kit (Tiangen, China) according to the manufacturer's protocol. For reverse transcription of RNA into cDNA, the iScript^TM^ gDNA Clear cDNA Synthesis Kit (Bio-Rad, USA) was used. The transcriptions were quantified using iTaq^TM^ universal SYBR Green supermix (Bio-Rad, USA). Primers were listed in Table S10. All qRT-PCR reactions were performed using an ABI 7500FAST Sequence Detector System (ABI, USA), and each gene expression was normalized with GAPDH mRNA content in the macrophages.

### In vitro anti-angiogenesis study

The ability of tube formation of the endothelial cells (HUVEC) in the presence of M1Φ or M2Φ was monitored according to a previous method 11. In contrast to the Transwell study above, the drugs were added to both upper and lower chambers to maintain the consistent drug concentration because the MΦ in upper chambers cannot form an intact monolayer, and drugs can freely diffuse through. The drugs (i.e., Gef 2 μg/mL, SV 2 μg/mL, or Gef/SV 2 μg/mL each) were added to both the chambers for 24 h to examine the ability of HUVEC tube formation.

### Antitumor activity

The anti-proliferation activity was evaluated by determining IC_50_ using MTT assay. A concentration of combo drugs means both drugs at such a concentration.

### Mechanism studies

The tumor cells were incubated with the liposomes (equal to 4 μg/mL of drugs) for 24 h, and subsequently, caspase-3, cleaved-caspase-3, or RIPA levels were examined using Western blotting. The expression of EGFR/Erk/Akt and their phosphorrylated forms, and the levels of NOX3/MrsA/GPX4/Bcl-2 were also measured by Western blotting after 48-h treatment.

The tumor cells were treated with the liposomes (equal to 4 μg/mL each) for 24 h, and the intracellular ROS production was determined using a reactive oxygen detection kit (Beyotime, Shanghai, China).

### In vitro macrophages re-education study

The M2Φ was treated with the free combo drugs (2 μg/mL) or each for 24 h, and then the M2-related biomarkers (e.g., CD206) were detected using Western blotting.

A Transwell system containing tumor cells in a lower chamber and M1Φ or M2Φ in the upper chamber was co-cultured for 24 h. The H1975 tumor cells were exposed to the drugs (4 μg/ml) while the M2Φ were cultured with the drugs (2 μg/ml) for 48 h. The EGFR/Erk/Akt levels were detected by Western blotting.

### The BM-bearing animal model

The BMs of lung cancer animal model was developed by intracranially implanting the H1975 NSCLC cells (2.5 × 10^5^) into the right striatum using a stereotaxic apparatus. The mice were used for further study at day 8 post-operation.

### Biodistribution study by in vivo imaging

The BMs-bearing mice were i.v. administered with the DIR-labeled liposomes (0.8 mg/kg). In vivo imaging was conducted using an IVIS system (Caliper PerkinElmer, Hopkinton, USA) at different time points. The disserted tumors and organs were subjected to ex vivo imaging at the endpoint. The tumors were then used for cryosection after fixation with 4% paraformaldehyde. The tissue sections were incubated with anti-PD-L1 antibody or TfR antibody, and subsequently with Cy3-conjugated secondary antibody, according to a normal Western blotting procedure. The fluorescence colocalization was observed by confocal laser scanning microscopy (TCS-SP8, Leica, Germany).

The circulation half-life measurement was executed to compare the Lipo and T12/P-Lipo labeled with Cy5.5. The female Balb/C mice were randomly divided into three groups (three mice per each group). Free Cy5.5, Lipo, and T12/P-Lipo were injected by tail vein injection with the same dose (equal to 2.5 mg/kg Cy5.5 dye for each mice). The mice were anesthetized and 0.2 mL of blood was collected by retro-orbital bleeding at predetermined time points. The plasma was obtained by centrifugation (5000 rpm, 10 min), and the Cy5.5 concentration was measured by a fluorescence spectrophotometer (F-4600, HITA-CHI, Japan). The half-life was calculated with DAS 2.0 PK software.

### In vivo therapeutic efficacy

The mouse model bearing BMs was developed as described above. The therapy study was performed 8 days after the intracranial inoculation of the H1975 cells. The animals were treated with Lipo, P-Lipo, or T12/P-Lipo at an i.v. dose equal to Gef/SV (8 mg/kg each) every 2 days (10 mice per group). Saline and Osi (8 mg/kg) were used as controls. The survival rate and animal bodyweight change were recorded to evaluate the anti-tumor therapeutic efficacy. The animals were euthanized when exhibiting signs of impaired motion or the bodyweight loss reached 30%. The tumor-bearing brains and organs were collected at the endpoint and weighed, and then fixed with 4% paraformaldehyde. The tumors-bearing brains were used for standard immunohistochemistry (IHC) staining to assess the anti-tumor efficacy (TUNEL assay, proliferation marker Ki67, and macrophages-associated markers CD206 or iNOS). Moreover, the tumor-bearing brains were also used for histopathological examination (HE staining) to assess the tumor regression.

### Western blotting assay

The Western blotting assay is briefly described as follows. The samples (cells or tissues) at the same protein concentration were loaded onto 12% SDS-PAGE gels and performed according to a standard protocol. After semi-dry transferring to a membrane, it was blocked with TBST buffer containing 5% BSA. Primary antibody incubation was performed at 4°C overnight to detect the targeted proteins and a housekeeping protein. After washing, the membranes were incubated with anti-rabbit IgG, HRP-linked antibody. After wash and a standard staining process, the membranes were used for digital imaging. The protein bands were semi-quantified using ImageJ, as shown in Table S3-7.

### Statistical analysis

All data were analyzed by GraphPad Prism software. The statistical analysis was performed by t-test and one-way ANOVA. Data were expressed as mean ± SD. Statistical difference was set as ^*^P<0.05, ^**^P<0.01, and ^***^P<0.001.
