# Supplementary material for: BBB-penetrating codelivery liposomes treat brain metastasis of non-small cell lung cancer with EGFRT790M mutation
Source: Theranostics. 2020 May 15;10(14):6122–35. doi: 10.7150/thno.42234 (PMC7255027; doi:10.7150/thno.42234)
Supplement: Supplementary file 1 — Supplementary figures and tables. [file thnov10p6122s1.pdf]

BBB-penetrating codelivery liposomes treat brain metastasis of non-small cell lung cancer with EGFR^{T790M} mutation

Weimin Yin [1, 2, #], Yuge Zhao [1, 3, #], Xuejia Kang [1], Pengfei Zhao [1], Xuhong Fu [1], Xiaopeng Mo [1], Yakun Wang [1], Yongzhuo Huang [1, 4, *]

Figure S1–S3

Table S1–S10

Figure S1 Characterization of DSPE-PEG$_{2000}$-T12 by H^{1}NMR and MALDI-TOF-Mass. (A) 1HMR of the DSPE-PEG$_{2000}$-T12. (B) The MALDI-TOF-Mass of the products DSPE- PEG$_{2000}$-T12.

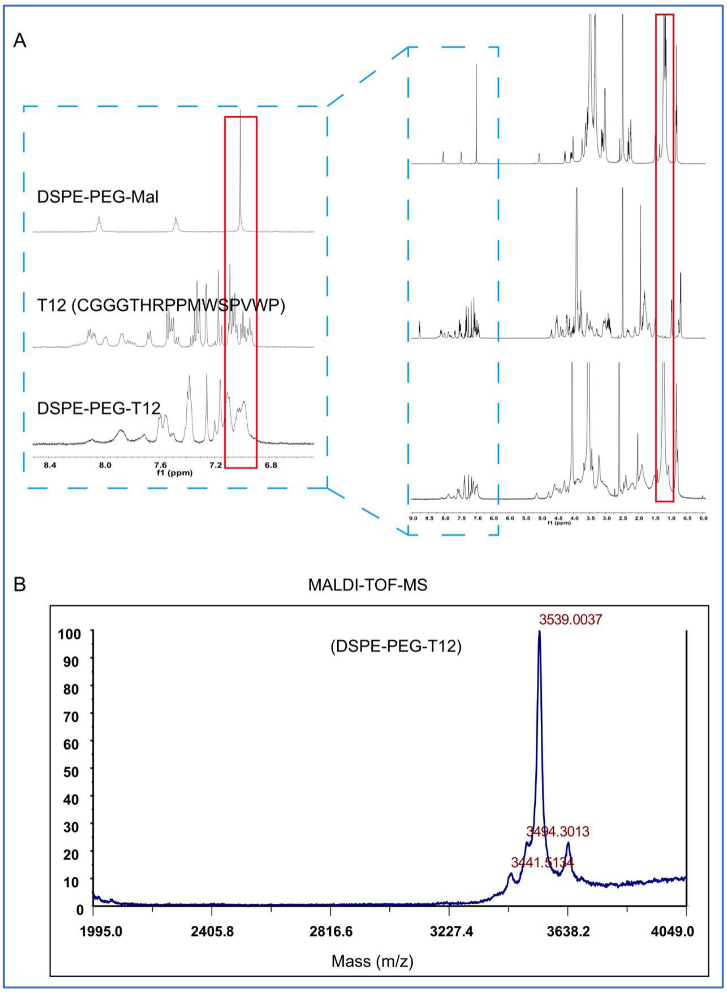

Figure S2 The characterizations of the liposomes. (A) The stability of the T12/P-Lipo. There was no significant change within 24 h. (B) In vitro drug release from the T12/P-Lipo in pH 6.5 medium, a mimic to tumor microenvironment. (C) The zeta potential of T12/P-Lipo. (D)The half-life of T12/P-Lipo and P-Lipo.

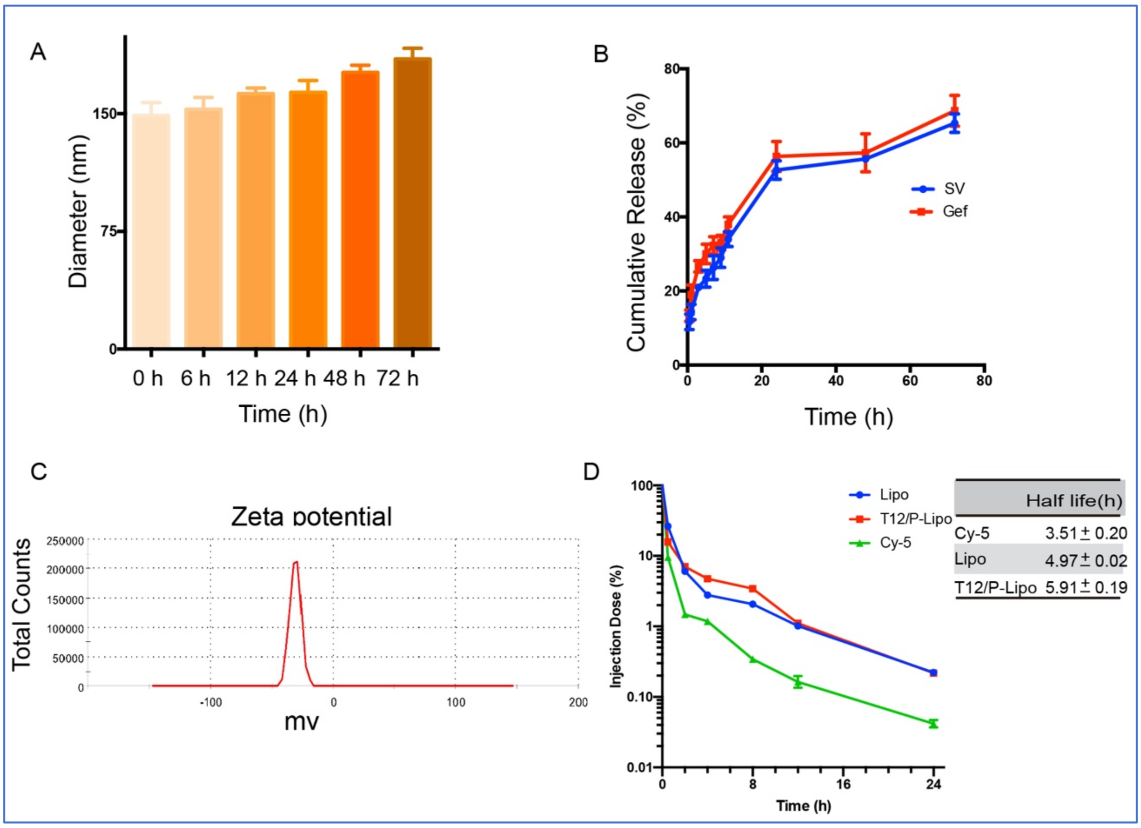

Figure S3 (A) The cell viability assessment of combination therapy Gef/SV in M1 or M2 macrophages. (B) The CD206 mRNA level expression of M2Φ after treatment via gradient ration of Gef/SV (1 μg/mL, 2 μg/mL, or 4 μg/mL), while M1 and M2Φ without treatment were set up as controls. (C) The cell viability assessment of Gef, SV or Gef/SV in HUVEC cells.

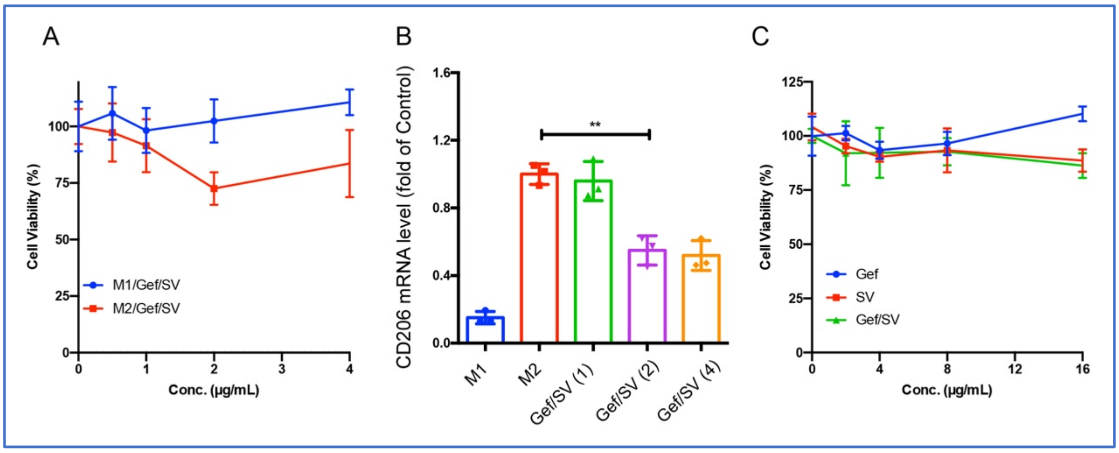

Table S1 Characterization of the liposomes

	size (nm)	PDI	DE (%) of Gef	DE (%) of SV	DL(%) of Gef	DL(%) of SV
Lipo	133 ± 4.08	0.15 ± 0.023	50.66 ± 4.5	75.33 ± 4.0	2.23 ± 0.40	1.86 ± 0.25
P-Lipo	142 ± 4.04	0.17 ± 0.021	52.00 ± 3.0	73.66 ± 4.7	2.13 ± 0.35	2.13 ± 0.25
T12/P-Lipo	153 ± 6.08	0.18 ± 0.010	51.33 ± 2.5	73.66 ± 3.1	2.46 ± 0.40	2.20 ± 0.30

Table S2 The relative levels of protein expression (GAPDH as a control)

	M1Φ	M2Φ	Gef	SV	Gef/SV
CD206	1.000	1.624	0.838	0.689	0.319
STAT1	1.000	0.911	1.256	1.292	4.252
GAPDH	1.000	1.000	1.000	1.000	1.000

Table S3 The relative levels of protein expression (GAPDH as a control)

	M1Φ	M2Φ	Gef	SV	Gef/SV	Osi	Lipo	P-Lipo	T12/P-Lipo
iNOS	1.000	0.079	0.058	0.042	0.037	0.035	0.028	0.049	0.055
CD206	1.000	5.212	9.238	9.609	9.638	7.320	7.185	6.865	9.810
TGF-β	1.000	1.7	2.049	1.358	1.759	1.412	1.798	1.469	0.865
GAPDH	1.000	1.000	1.000	1.000	1.000	1.000	1.000	1.000	1.000

Table S4 The relative levels of protein expression (GAPDH as a control)

	H1975 alone	M1Φ	Co-culture with M2Φ							
	Blank	Blank	Blank	Gef	SV	Gef/SV	Osi	Lipo	P-Lipo	T12/P-Lipo
EGFR	1.000	0.980	0.856	0.476	0.546	0.538	0.333	0.790	0.615	0.367
p-EGFR	1.000	0.715	1.430	0.743	0.817	0.763	0.312	0.792	0.600	0.520
Akt	1.000	1.383	1.092	1.006	1.003	0.794	0.567	0.576	0.476	0.398
p-Akt	1.000	1.303	1.828	0.633	1.219	0.704	0.709	0.688	0.625	0.580
Erk	1.000	1.571	0.756	0.673	0.673	0.754	0.667	0.870	0.791	1.010
p-Erk	1.000	0.993	1.020	0.939	1.192	0.890	0.530	0.611	0.533	0.340
MrsA	1.000	1.195	1.788	0.948	0.8654	0.546	0.325	0.458	0.417	0.068
GAPDH	1.000	1.000	1.000	1.000	1.000	1.000	1.000	1.000	1.000	1.000

Table S5 The relative levels of protein expression (GAPDH as a control)

	Blank	Gef	SV	Gef/SV	Osi	Lipo	P-Lipo	T12/P-Lipo
EGFR	1.248	1.605	1.159	0.9807	1.070	1.141	0.8559	0.7727
p-EGFR	1.070	1.010	1.212	0.8559	0.2675	0.6241	0.4279	0.2140
Akt	1.046	1.111	0.9510	0.8321	0.5825	0.6241	0.4993	0.4993
p-Akt	0.8920	0.8024	0.7132	0.5349	0.4279	0.4993	0.5825	0.4577
Erk	0.9510	0.8024	0.6657	0.7489	0.6241	0.6657	0.6186	0.4636
p-Erk	0.6180	1.010	0.6657	0.5349	0.3566	0.4636	0.4160	0.5349
MsrA	0.4990	0.6241	0.4993	0.5825	0.4636	0.6657	0.5706	0.4993
GAPDH	1.177	0.9510	1.010	0.9510	1.141	0.7489	0.951	1.070

Table S6 The relative levels of protein expression (GAPDH as a control)

	Blank	Gef	SV	Gef/SV	Osi	Lipo	P-Lipo	T12/P-Lipo
NOX3	1.000	1.397	3.636	3.563	2.351	3.244	4.630	5.938
MrsA	1.000	0.880	1.356	1.164	1.075	1.045	0.921	1.164
GPX4	1.000	1.118	1.240	0.819	0.611	1.080	1.089	0.819
Bcl-2	1.000	0.782	1.045	0.970	0.576	0.625	0.643	0.600
GAPDH	1.000	1.000	1.000	1.000	1.000	1.000	1.000	1.000

Table S7 The relative levels of protein expression (GAPDH as a control)

	Blank	Gef	SV	Gef/SV	Osi	Lipo	P-Lipo	T12/P-Lipo
Caspase-3	1.000	0.933	0.869	0.600	0.767	0.539	0.56	0.427
Cleaved Caspase-3	1.000	1.333	1.255	1.560	2.175	2.274	2.38	3.360
GAPDH	1.000	1.000	1.000	1.000	1.000	1.000	1.000	1.000

Table S8 Statistical analysis of survival curve of the animals with H1975 intracranial tumor

	PBS		Gef		Gef/SV		Lipo		P-Lipo		T12/P-Lipo		Osi	
	Chi-Square	P-Value	Chi-Square	P-Value	Chi-Square	P-Value	Chi-Square	P-Value	Chi-Square	P-Value	Chi-Square	P-Value	Chi-Square	P-Value
PBS			1.272	0.2593	3.664	0.0556	4.510	0.0337	15.36	<0.0001	18.15	<0.0001	20.41	<0.0001
Gef	1.272	0.2593			1.322	0.2503	2.250	0.1336	10.92	0.0010	14.18	0.0002	18.06	<0.0001
Gef/SV	3.664	0.0556	1.322	0.2503			0.2924	0.5887	5.376	0.0204	10.11	0.0015	13.68	0.0002
Lipo	4.510	0.0337	2.250	0.1336	0.2924	0.5887			3.747	0.0529	8.251	0.0041	12.41	0.0004
P-Lipo	15.36	<0.0001	10.92	0.0010	5.376	0.0204	3.747	0.0529			1.834	0.1757	4.155	0.0415
T12/P-Lipo	18.15	<0.0001	14.18	0.0002	10.11	0.0015	8.251	0.0041	1.834	0.1757			0.7212	0.3958
Osi	20.41	<0.0001	18.06	<0.0001	13.68	0.0002	12.41	0.0004	4.155	0.0415	0.7212	0.3958		

Table S9 The changes of each group after treatment

	PBS	Gef	Gef/SV	Lipo	P-Lipo	T12/P-Lipo	Osi
0 day	10	10	10	10	10	10	10
8 day	10	10	10	10	10	10	10
10 day	9	10	10	10	10	10	10
12 day	8	9	10	10	10	10	10
14 day	7	8	10	9	10	10	10
16 day	5	7	7	9	10	10	10
18 day	3	5	6	7	10	10	10
20 day	2	4	5	6	9	10	10
22 day	1	2	4	4	8	9	10
24 day	0	1	4	4	7	8	9
26 day	0	0	2	3	5	8	8
28 day		0	0	1	5	6	8
30 day			0	0	3	5	7
32 day				0	2	4	6
34 day					2	4	5
36 day					1	3	3
38 day					0	2	3
40 day					0	1	2
42 day						0	1

Table S10 The primer sequence of genes

Gene	Forward primer	Reverse primer
CD206	TCTTTGCCTTTCCCAGTCTCC	TGACACCCAGCGGAATTTC
TNF-alpha	CCTGTAGCCCACGTCGTAGC	AGCAATGACTCCAAAGTAGACC
GAPDH	TATGTCGTGGAGTCTACTGGT	GAGTTGTCATATTTCTCGT
